# Neural encoding of auditory rhythm beyond cortical auditory areas before the age of term

**DOI:** 10.1016/j.isci.2025.113028

**Published:** 2025-07-24

**Authors:** Ali Rajabi Mashhadi, Fabrice Wallois, Mohammadreza Edalati, Florence Levé, Alexandros Stamatiadis, Christelle Chazal, Laurel Trainor, Sahar Moghimi

**Affiliations:** 1 Inserm UMR1105, GRAMFC, Université de Picardie Jules Verne, Amiens, France; 2 Inserm UMR1105, EFSN Pédiatriques, CHU, Amiens, France; 3 MIS, Université de Picardie Jules Verne, Amiens, France; 4 University Lille, CNRS, Centrale Lille, UMR 9189 CRIStAL, 59000 Lille, France; 5 McMaster Institute for Music and the Mind, McMaster University, Hamilton, ON L8S 3L8, Canada; 6 Rotman Research Institute, Baycrest Hospital, Toronto, ON M6A 2E1, Canada

**Keywords:** Cognitive neuroscience, Neuroscience, Sensory neuroscience

## Abstract

Rhythm experience begins in fetal life, shaping neural capacities critical for language, communication, and motor skills. While rhythm processing in adults involves distributed cortical networks, including premotor and supplementary motor regions, the mechanisms in the fetal brain remain unclear. We provide evidence that premature newborns encode rhythmic beats through cortical networks extending beyond the auditory cortex into premotor and sensorimotor regions. Using high-density functional near-infrared spectroscopy, we show that auditory beats trigger distinct cortical activation patterns, indicating early involvement of an auditory-motor network, despite the absence of coordinated motor activity. Our results highlight a fundamental role for these regions in rhythm perception, forming the basis for predictive timing mechanisms. This early engagement of sensorimotor regions reveals a neural framework supporting beat perception from the fetal stage onward. These findings advance understanding of the neural architecture for rhythm processing, showing that the premature brain is already wired for complex auditory-motor interactions.

## Highlights

• We presented regular and irregular auditory rhythms to sleeping preterm newborns
• We used HD-fNIRS to map local hemodynamic responses across multiple channels
• Non-isochronous rhythms enhanced cortical activity beyond auditory into motor regions
• Sensorimotor regions are already involved in beat processing before full term

## Graphical abstract

**Figure:**
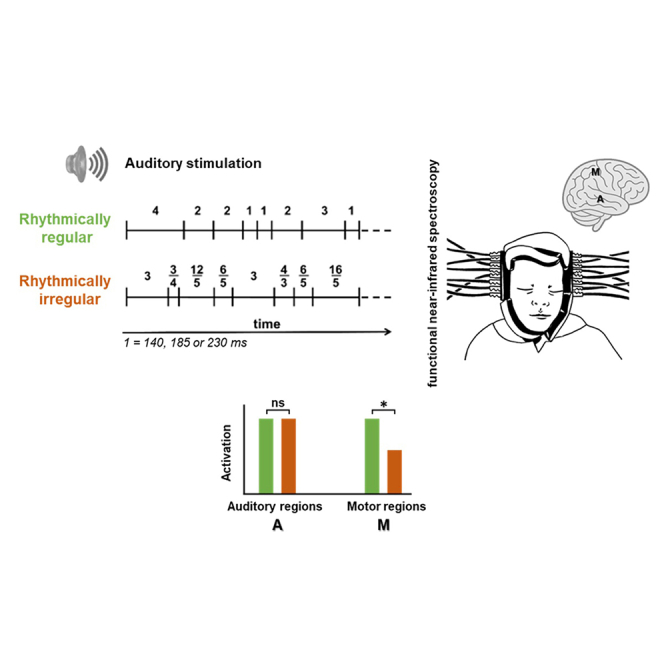

## Introduction

Auditory rhythm experience begins early in human life, from the late second trimester of gestation.^1^^,^^2^ As the fetal auditory system matures, the unborn child is exposed to a symphony of rhythmic sounds, from the omnipresent maternal heartbeat to the nuanced maternal and exogenous rhythms of speech and music transmitted through maternal tissue. This early exposure is not merely incidental; rather, it is a cornerstone of neurodevelopment. Emerging evidence suggests that rhythm-processing abilities extend far beyond musicality, serving as foundational pillars for language acquisition, communication, social interactions, and even motor coordination.^3^^,^^4^^,^^5^^,^^6^^,^^7^

When adults listen to a typical musical rhythm, they can spontaneously extract and predictively entrain to its underlying beat.^8^^,^^9^^,^^10^^,^^11^ That the perceived beat is derived in the brain is evident in that beats can be perceived even during rests (or silences) in a repeating rhythmic pattern and when there is little energy at the beat frequency in the stimulus.^12^^,^^13^ It is hypothesized that sensorimotor mechanisms play an important role in the perception of musical beat.^14^^,^^15^^,^^16^ In this line, neuroimaging evidence in human adults suggests that musical and speech rhythm perception elicits activity across cortical premotor and sensorimotor areas, even when no overt movement is involved.^17^^,^^18^^,^^19^^,^^20^^,^^21^^,^^22^^,^^23^^,^^24^^,^^25^ There is also evidence for auditory-motor interactions during rhythm processing in infancy, with bouncing patterns impacting the perceived rhythmic structure in 7-month-olds.^26^ However, how early auditory-motor interactions are present is not known; nor are the underlying neural mechanisms for early rhythm perception clear.

**Figure 1:**
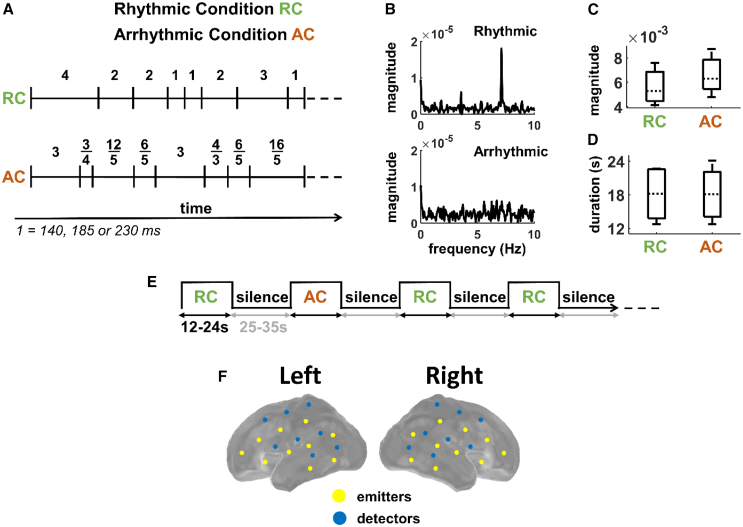
Experimental design (A) Schematic representation of a sample rhythmic sequence and its arrhythmic counterpart with timing of intervals. Vertical bars indicate interval onset. (B) Corresponding power spectra of the rhythmic and arrhythmic sequences in (A), showing the absence of beat frequencies in the arrhythmic case. (C and D) Comparison of envelope power (C) and duration (D) of stimuli between rhythmic and arrhythmic sequences. Error bars represent standard deviation. (E) The block-designed protocol. Rhythmic condition (RC) and arrhythmic condition (AC) trials were presented in pseudo-random order (see text). (F) Schematic representation of the emitters and detectors position on the brain of a 35 wGA preterm infant.

The full-term brain and the near-term brain encode the beat in rhythmic sequences^27^^,^^28^ and even show evidence for predictive timing.^29^^,^^30^^,^^31^ These neural capacities develop progressively during the third trimester of gestation,^32^ laying the groundwork for the complex interplay between auditory and motor systems that will support language and music learning throughout life.^33^^,^^34^ What are the cortical structures underlying very early rhythm processing and encoding? Does this early neural capacity to track rhythmic progressions rely solely on auditory networks or does it involve distributed cortical/neural structures including sensorimotor and premotor regions as in adults?^35^^,^^36^ The motor and premotor regions are immature during the third trimester of gestation, and coordinated body movement is far from being established. It takes several months for infants to begin spontaneously engaging in rhythmic movement.^37^ Therefore, early activation of motor-related regions during auditory rhythm perception would suggest that that these regions are recruited for rhythm processing prior to substantial involvement in motor pattern execution. Here, we asked whether rhythm perception in the premature brain is an early manifestation of the same sophisticated neural circuitry that adults use to process complex auditory patterns. To answer these questions, we presented auditory sequences that were rhythmically regular or rhythmically irregular (Figure 1A; see STAR Methods for details) to sleeping premature newborns while measuring cortical activity. Specifically, we tested whether disrupting the rhythmicity of auditory sequences alters activation patterns in the dorsal and ventral auditory pathways. Using high-density functional near-infrared spectroscopy, we mapped local hemodynamic responses across multiple channels, providing a comprehensive view of cortical activation patterns. We hypothesized that if a salient and regular beat is encoded across distributed cortical regions, disrupting the rhythmicity—while maintaining the overall envelope energy and duration (Figures 1C and 1D)—would trigger distinct activation patterns in brain regions beyond the auditory cortex.

## Results

### Hemodynamic response to auditory stimulation

**Figure 2:**
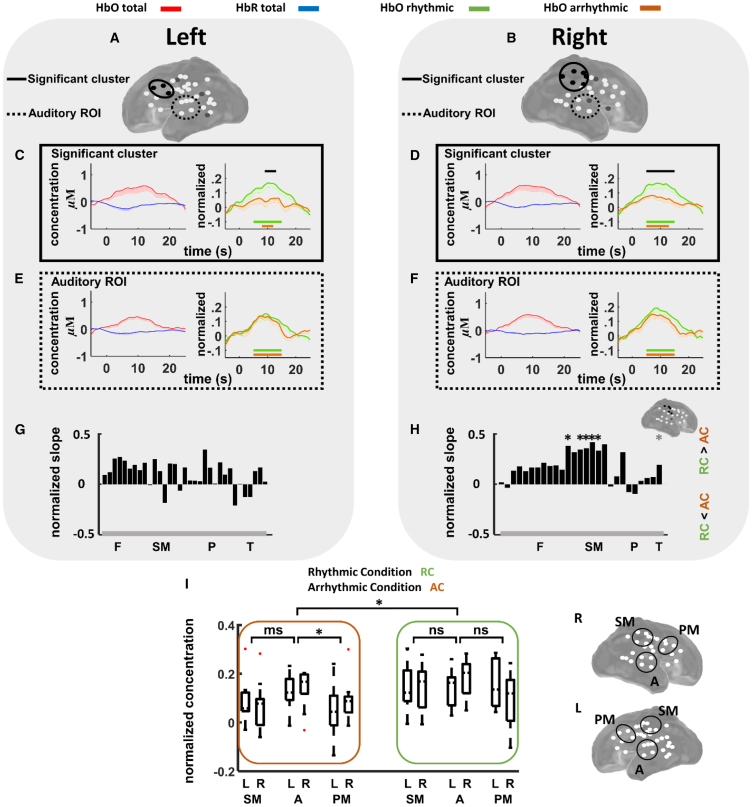
Hemodynamic responses to rhythmic and arrhythmic conditions over different cortical regions (A and B) Estimated projection of the remaining channels after preprocessing (white circles) over the left (A) and right (B) hemispheres. Channels with significant differences in the HbO response to rhythmic versus arrhythmic conditions are shown in gray circles for before and in black circles for after correction for multiple comparisons. (C–F) Neurovascular coupling for all trials (left) and differences in rhythmic and arrhythmic HbO responses (right) over significant clusters (C and D, marked by continuous black line in A and B) and auditory regions (E and F, marked by dashed black line in A and B), where there were no significant differences between rhythmic and arrhythmic conditions. The black bars indicate the time windows during which the rhythmic condition (RC) differs significantly from the arrhythmic condition (AC). The green bars indicate the time windows during which the RC differs significantly from its baseline, whereas the orange bars indicate the time windows during which the AC differs significantly from its baseline. Curves are represented as mean (−SEM). (G and H) Rising slope of HbO responses over the left (G) and right (H) hemispheres. The normalized slope is calculated as (Slope_RC_ − Slope_AC_)/(Slope_RC_ + Slope_AC_) (∗: significant difference before [gray] and after [black] correction). Inset shows the position of channels belonging to the significant cluster (black circles). F, frontal; SM, sensorimotor; P, parietal; T, temporal channels. Cortical division of channels is approximate and for visual representation. (I) Comparison of average HbO responses (8–12 s) over sensorimotor, auditory, and premotor ROIs. ∗: significant difference (p < 0.05), ms: marginally significant difference (0.05<p<0.1), ns: non-significant difference (p > 0.1). L, left; R, right; SM, sensorimotor; A, auditory; PM, premotor. Error bars represent standard error.

We first evaluated the neural response to auditory stimulation. The typical neuro-vascular coupling (HbO increase and HbR decrease) previously observed in premature infants^38^^,^^39^ emerged globally in response to auditory stimulation with a time course similar to that of full-term infants and adults, as shown in the grand average response calculated over all the trials (rhythmic and arrhythmic) (Figures 2C–2F; see also Figure S1 for each channel separately). The cluster-based analyses, separately comparing the HbO response for each condition with baseline, both revealed temporal clusters over a large number of channels (Figures S1 and 2), indicating that both conditions elicited a hemodynamic response. Precisely, rhythmic sequences elicited a significant increase in HbO compared to baseline in 25 channels over the right hemisphere (out of 27) and in 28 channels over the left hemisphere (out of 33), whereas arrhythmic sequences elicited a significant increase in HbO compared to baseline in 17 channels over the right hemisphere and in 25 channels over the left hemisphere (Figure S1).

### The impact of rhythmicity on the hemodynamic response

We then investigated the neural activity specific to rhythmicity. Toward this, we contrasted the HbO response to rhythmic sequences with that corresponding to arrhythmic sequences. The hemodynamic response was faster and larger over the right sensorimotor and left premotor regions. More precisely, two significant clusters emerged over the left premotor cortex (*p*_cluster_ = 0.038) and the right sensorimotor cortex (*p*_cluster_ = 0.012) around the peak of the hemodynamic response, which was at ∼10 s (Figures 2A and 2B), due to relatively higher hemodynamic activity in response to the rhythmic condition compared to the arrhythmic condition over these regions. Interestingly, no significant cluster emerged over the auditory regions, where visual inspection of the average HbO responses also revealed relatively similar amplitudes for the two conditions (Figures 2E and 2F), which had relatively similar sound energy. The rising slope of the HbO response to rhythmic sequences was significantly larger than that corresponding to arrhythmic sequences over the right hemisphere in regions beyond the auditory cortex (Figures 2G and 2H), suggesting a higher metabolic demand from the earliest seconds of the rhythmic sequences over this region. Spatial permutation test revealed a significant cluster (*p*_cluster_ = 0.010) over the right sensorimotor regions.

### Comparing auditory and sensorimotor regions

To further investigate cortical activation beyond the auditory cortex and notably over sensorimotor and premotor regions due to rhythmic regularities, we defined three focalized regions of interest (ROIs), one over the auditory cortices, one over the sensorimotor regions, and one over the premotor regions in both hemispheres (Figure 2I inset), and then examined the average HbO response in the window 8–12 s. Three-way repeated ANOVA (rANOVA) with factors condition (rhythmic, arrhythmic), ROI, and hemisphere revealed a significant effect of condition (F(1, 10) = 7.854, *p* = 0.021, *η*_*p*_^2^ = 0.466), a significant effect of ROI (F(2, 20) = 4.804, *p* = 0.033, *η*_*p*_^2^ = 0.348), and a significant effect for the interaction condition × ROI × hemisphere (F(2, 20) = 9.849, *p* = 0.003, *η*_*p*_^2^ = 0.523). The factor hemisphere and the interactions condition × ROI, ROI × hemisphere, and condition × hemisphere did not show significant effects. In follow-up analyses, separate rANOVAs for each of the rhythmic and arrhythmic conditions with factors ROI and hemisphere revealed a significant effect of ROI (F(2, 20) = 8.662, *p* = 0.006, *η*_*p*_^2^ = 0.490) in the arrhythmic condition, with larger cortical activity over the auditory cortex compared to the sensorimotor (*p* = 0.056; marginally significant) and premotor (*p* = 0.014; significant) regions but no significant effect of ROI in the rhythmic condition (Figure 2I). No significant effect was found for the factor hemisphere or the interaction ROI × hemisphere. Finally, separate rANOVAs for each of the three ROIs with factors condition and hemisphere revealed a significant effect of condition for the sensorimotor ROI (F(1, 10) = 11.762, *p* = 0.006, *η*_*p*_^2^ = 0.541) and a marginally significant effect of condition for the premotor ROI (F(1, 10) = 4.521, *p* = 0.059, *η*_*p*_^2^ = 0.311), with larger activity over the sensorimotor and premotor regions in the rhythmic condition compared to the arrhythmic condition, and no significant difference between conditions for the auditory ROIs. The interaction condition × hemisphere revealed a significant effect only for the premotor ROI (F(1, 10) = 19.779, *p* = 0.001, *η*_*p*_^2^ = 0.664). Put together, these analyses revealed that (1) cortical activation was larger over auditory than sensorimotor and premotor regions in the arrhythmic condition but did not differ between regions in the rhythmic condition, and (2) in the sensorimotor and premotor regions, cortical activity was larger in the rhythmic relative to the arrhythmic condition, whereas in the auditory regions, cortical activation was similar across rhythmic and arrhythmic conditions.

## Discussion

Increasing evidence suggests that the motor system is involved in encoding the beat,^14^^,^^25^ engaging structures along the dorsal auditory pathway^9^^,^^14^ in generating temporal predictive oscillations, in interaction with auditory regions.^36^^,^^40^^,^^41^ The majority of this evidence is from human adults, where studies show that even in the absence of movement, different regions of the motor system are active and likely *necessary* for beat perception. However, many studies now show that young infants’^42^^,^^43^ and even premature newborns’ brains^28^^,^^29^ track both beat and metrical structure in rhythms. Similarly, despite the theory that the perception of speech involves a motor representation of how to articulate the speech (motor theory of speech perception^17^), newborns discriminate phonemes despite being unable to produce them.^39^^,^^44^ These early abilities thereby raise as puzzle because the motor system is quite immature early in development, and it takes some time before children can easily coordinate synchronized movement to auditory rhythms.^45^^,^^46^^,^^47^ At the same time, while rhythmic execution requires primary motor movements, there is behavioral evidence suggesting that premotor or supplementary motor regions may already be interacting during beat and meter processing in infancy.^26^ This raises the question as to what the underlying mechanisms are and the engaged cortical structures for beat perception during early neurodevelopment, prior to the emergence of rhythmic movement and maturation of the motor system. Here, we presented the first evidence for the early involvement of premotor and sensorimotor regions in beat perception before the age of term. We showed that exposure to non-isochronous rhythms with stable beats leads to enhanced activity in the cortical regions of the auditory pathway dorsally beyond the auditory cortex and into the sensorimotor and premotor regions. Precisely, neural activity over motor areas in response to rhythmic stimuli was larger than that in response to controlled arrhythmic stimuli, whereas neural activity over the auditory areas did not differ between rhythmic stimuli compared to controlled arrhythmic stimuli. We also showed that the neural response dynamic was faster over motor areas in response to rhythmic stimuli compared to controlled arrhythmic stimuli.

Stable overt motor synchronization to rhythm does not arrive before 3–4 years of age.^48^ However, home studies show that moving to music develops early, with a clear improvement over the first 2 years after birth.^37^ Spontaneous periodic movements to music are observed in infants beginning from 5 to 6 months of age.^49^^,^^50^ There is even evidence that infants as young as 3–4 months can occasionally exhibit rhythmic movement in response to music.^51^ Interestingly, passive body movement (being bounced) influences how 7-month-olds perceive the structure of an auditory rhythm,^26^ suggesting that passive movement synchronization also contributes to infants’ encoding of auditory rhythms. Indeed, young infants spend much of their day experiencing the rhythmic movements of their caregiver as they are held, rocked, bounced, and carried. Prenatally, in addition to different rhythmic sounds, from the omnipresent maternal rhythms to the nuanced patterns of maternal and external rhythms of speech and music, the fetus also experiences rhythmic movement as the mother moves. Our findings suggest a fundamental role for the premotor and sensorimotor regions in auditory beat perception long before the emergence of overt movement, let alone movement synchronization, suggesting that these regions play a fundamental early role in a neural network spanning auditory and motor cortical areas. What might be the role of the sensorimotor regions in early beat processing? There is electroencephalogram (EEG) evidence for predictive rhythmic processing in near-term and full-term newborns^28^^,^^31^ and young infants.^42^^,^^43^ However, EEG does not allow reliable localization of cortical sources and detection of the underlying cortical regions, particularly in cases of multiple active sources.^52^^,^^53^ Therefore, until now, we could not test hypotheses regarding the underlying neural structures and their possible role in rhythm processing during very early development. That the sensorimotor and premotor regions show enhanced activation in the presence of a salient beat in premature infants (with a regularity that enables prediction of the next beat), together with previous findings for predictive beat processing at this stage of neurodevelopment,^29^ suggests the possible presence of an immature version of beat-based predictive timing in an auditory-motor network, similar to that depicted in adults.^20^^,^^21^ In addition, the observed activity over sensorimotor and premotor regions may be precursors of activities observed in adults over motor planning regions while listening to music without overt movement involved,^18^^,^^23^ and related to a premature and uncoordinated version of motor timing, that will later shape into early spontaneous movement to the musical beat during infancy.

The tendency we found for faster right- than left-lateralized activation might be related to general auditory development as previous studies have reported a right-sided lateralization to auditory syllables in premature newborns^39^^,^^54^ and to pitch sequences in young infants.^55^ The right hemispheric asymmetry in premotor and sensorimotor activation might suggest an early hemispheric differentiation in coding temporal regularities that may evolve with neurodevelopment, since this lateralization is not in line with left-lateralized observations over sensorimotor regions in rhythm perception in adults.^20^^,^^56^ Future studies are needed to investigate the developmental evolution of lateralization for rhythm processing in association with neural structural development.^57^^,^^58^

In the newborn brain, primary sensorimotor cortices mature before associative cortex,^59^^,^^60^ and corticomuscular communication is already functional during the late prenatal stage.^61^ In addition, electrical activity related to sporadic movement—potentially triggered by spontaneous activity in the spinal cord, brainstem, or primary motor cortex^62^—plays an important role in early developmental physiological processes and has been recorded over the motor cortex as young as 28 wGA.^63^ Furthermore, the dorsal fiber tract from the temporal cortex to the premotor cortex is already myelinated at birth,^64^^,^^65^ highlighting the early functional role of these networks that are crucial for future auditory-motor integration. However, the network is immature in infants. Adult models of beat perception based on neurophysiological and behavioral data suggest top-down modulation of activity in auditory cortex from motor areas related to temporal prediction.^9^^,^^21^^,^^66^ In addition, children with motor deficits show impairments in auditory rhythm and timing processing,^67^ providing indirect evidence for the influence of motor networks on auditory temporal processing. Consistent with a bi-directional model, adults show enhanced activity over the auditory regions in addition to the premotor and supplementary motor areas in response to rhythmic compared to arrhythmic stimuli.^19^ The current study does not allow directly addressing the causality and direction of modulation between sensorimotor and auditory regions in premature infants. However, given that our stimuli were presented through the auditory modality, in conjunction with our finding that rhythmicity modulated activity over sensorimotor regions but not over the auditory regions, these together suggest stronger pathways from auditory to motor regions than vice versa. Specifically, motor regions were sensitive to rhythmicity, but this did not translate back to auditory regions. This might reflect immature or weak top-down modulation of efferent activity from premotor and motor regions to the auditory cortex, although these pathways have not been directly studied before the age of term. Of course, these interpretations are highly hypothetical. A cross-sectional study during early development would allow investigating how cortical beat encoding matures with the development of the motor system and the long-range intracortical connections.

An advantage of our study was the use of high-density optode patches, allowing replication of the observed differences over neighbor channels. While the quality of source localization in functional near-infrared spectroscopy in adults is comparable to that obtained with functional magnetic resonance imaging,^68^ provided that a high sensor density is implemented, there is currently no data available for premature newborns. However, even considering the uncertainties regarding the scattering and absorption coefficients of neonatal tissues in premature neonates, as well as the impact of fontanels, it is unlikely that the localization error exceeds a few millimeters.^69^ By utilizing an age-matched head model with the digitization of the emitters and detectors, we are confident in our definition of the motor and sensorimotor regions, as this high-density setup allows us to visualize changes across three neighboring channels.

It should also be noted that our neural recordings were taken while premature newborns were sleeping. While this might limit comparisons between infant and adult studies, a significant distinction exists between adults and neonates in their capacity to perform complex neural coding during sleep. Sleeping newborns exhibit neural capacities for encoding aspects of the auditory environment—particularly related to music and speech—that are less pronounced in adults during sleep.^31^^,^^70^^,^^71^^,^^72^^,^^73^ These differences may stem from variations in the organization of sleep-wake structures and mechanisms, as well as the distinct influence of subcortical sleep regulators (see discussion in Saadatmehr et al.^32^). This leads to the hypothesis that cortical encoding of external information in fetuses and neonates may rely on different neural regions compared to later developmental stages. Furthermore, it is possible that learning and consolidation during sleep are more efficient in early life than in later stages of development.

In conclusion, the current study is novel in showing that the presentation of auditory rhythmic stimuli activates sensorimotor regions in sleeping premature newborns differently than arrhythmic stimuli, demonstrating that, surprisingly, sensorimotor regions are involved in beat processing during very early neurodevelopment, at least a month before full term. These results highly encourage further multimodal studies that would allow drawing conclusions on the bottom-up and top-down roles of the developing dorsal auditory pathway in rhythm processing during early development. This neurodevelopmental model will help in understanding the neural building blocks of rhythm processing. Learning in neural networks needs time^74^ and depends on development and expertise.^75^ Many questions remain, and future studies should compare how neural activation beyond auditory cortices is affected by factors, such as the complexity of rhythmic structure, across early development.^15^^,^^25^^,^^76^

### Limitations of the study

A limitation of our study was that the use of fiber optic patches did not allow us to record the supplementary motor area and the more dorsal regions of the motor system at the same time as the ventral parts. Therefore, we could not evaluate the findings regarding the role of the supplementary motor areas in beat encoding^19^^,^^22^^,^^77^ at this stage of neurodevelopment. Further studies with more extensive recording sites are necessary to better understand the role of these regions in rhythm processing across infant development. Another limitation of the current study is related to the small number of premature neonates. However, we successfully replicated the auditory hemodynamic response pattern from our previous studies,^39^ which supports the reliability of our findings despite the modest sample size. Finally, due to the relatively small sample size, we did not investigate the impact of sex on the obtained results. This effect needs to be evaluated in future studies.

## Resource availability

### Lead contact

Further information and requests for resources, data, and codes should be directed to and will be fulfilled by the lead contact, Dr. Sahar Moghimi (sahar.moghimi@u-picardie.fr).

### Materials availability

All materials and methods are presented in the paper, are available on GitHub (https://github.com/mredalati/Stimuli-for-fNIRS-recording), or can be made available upon request from the lead contact.

### Data and code availability

• Data: the raw data investigated in the current manuscript are privileged patient data. Due to parents’ non-consent to share their data beyond our research consortium, the data are not publicly accessible as per the consent form. Access will be granted to named individuals in accordance with ethical procedures governing the reuse of clinical data, including completion of a formal data sharing agreement.
• Code: the MATLAB code along with preprocessed data necessary to reproduce the figures and results is available on GitHub (https://github.com/mredalati/Stimuli-for-fNIRS-recording).
• Additional information: any additional information that is required to analyze the data reported in the paper can be obtained by contacting the lead contact.

## Acknowledgments

This work has received funding from the European Union’s Horizon 2020 research and innovation program under grant agreement no. 101017113. This work was also supported by Fondation pour l’Audition Grant BabyMusic RD-2021-11 and Agence TNationale de la Recherche Grant ANR-22-CE37-0032.

## Author contributions

Study conception and design, F.W., F.L., A.S., L.T., and S.M.; data collection, A.R.M.; data processing and interpretation of results, A.R.M., F.W., M.E., L.T., and S.M.; draft manuscript preparation, A.R.M. and S.M.; revisions, A.R.M., F.W., F.L., L.T., and S.M. All authors reviewed and approved the final version of the manuscript.

## Declaration of interests

The authors declare no competing interests.

## Declaration of generative AI and AI-assisted technologies in the writing process

During the preparation of this work, the authors did not use generative AI and AI-assisted technologies.

## STAR★Methods

### Key resources table

**Table:** 

REAGENT or RESOURCE	SOURCE	IDENTIFIER
**Software and algorithms**		

MATLAB R2022b	MathWorks	https://www.mathworks.com/products/matlab.html
FieldTrip	Oostenveld et al.^78^	https://www.fieldtriptoolbox.org/
Psychtoolbox	N/A	http://psychtoolbox.org/
Homer3	Huppert et al.^79^	https://github.com/BUNPC/Homer3/wiki
AtlasViewer	Aasted et al.^80^	https://github.com/BUNPC/AtlasViewer/wiki

**Other**		

Stimuli	This paper	https://github.com/mredalati/Stimuli-for-fNIRS-recording
Code	This paper	https://github.com/mredalati/Stimuli-for-fNIRS-recording

### Experimental model and study participant details

#### Participants

Thirteen healthy preterm neonates (6 females, mean gestational age at birth, 31.6 ± 2.9 wGA) participated in this study after obtaining the written consent form. All neonates were tested during sleep (mean recording age, 36.1 ± 1.5 wGA). All neonates had appropriate birth weight, size, and head circumference for their gestational age and normal auditory and clinical neurological assessments (Table S1). None were considered to be at risk of brain damage. One or both parents were informed about the study and provided their written informed consent. The study was approved by the local ethics committee (Comité Pour la Protection des Personnes Nord-Ouest I; ID-RCB: 2021-A02556-35).

### Method details

#### Stimuli

Five rhythmic (i.e., beat-based) sequences of tones were created, using methods adapted from.^73^ Four related onset-to-onset durations (i.e., intervals) between successive tones occurred in the sequences. In Table S2 and Figure 1A, the fastest interval is represented by 1 time unit, with the other intervals being two, three, and four times the duration of 1 time unit, that is being 2, 3, and 4 time units, respectively. The five sequences consisted of between 43 and 46 tones (i.e., 42–45 intervals). We used Ableton Live to create percussion sounds by the superposition of woodblock and piano timbres, with superposition being controlled using SSL Bus Compressor with the highest attack and lowest release settings and 2:1 compression Ratio. WAV sound files were created for each rhythmic sequence at three tempi (interval 1 = 140, 185 and 230 ms) and two piano pitches (880Hz and 440 Hz), resulting in a total of 30 sequences whose durations varied between 13 and 24 s. All the rhythmic sequences are provided as supplementary material (Table S2, WAV files in github.com/mredalati/Stimuli-for-fNIRS-recording).

For each of the five rhythmic sequences, a corresponding arrhythmic sequence was created using music21 toolbox.^79^ To accomplish this, a random one-third of the intervals in each interval category (i.e., 1, 2, 3, 4) in the regular sequence were randomly shortened by 20%, 25%, or 33%, one-third remained the same, and one-third were lengthened by 20%, 25%, or 33%. All the intervals were then randomly shuffled (Figure 1A; Table S2). This strategy destroyed the rhythmic regularities (see Figures 1B and S2), while keeping the number of intervals identical, and the overall sequence length and envelope power very similar (Figures 1B–1D). As with the rhythmic sequences, 30 WAVE files were synthesized crossing the 5 sequence, 3 tempi and 2 pitches. In order to estimate the temporal envelope of the stimuli, the absolute value of the Hilbert transform was computed for each sound signal. Using a fast Fourier transform, we then averaged each resulting spectrum from 0 to 10 Hz to compute the average power of each stimulus. A paired sample t-test revealed no significant difference between the duration and frequency power (<10 Hz) of the rhythmic and arrhythmic sequences (*p* > 0.05).

#### Experimental paradigm and data recording

60 blocks (i.e., the 30 rhythmic and 30 arrhythmic sequences) were played for each infant in random order with the constraints that no more than two rhythmic or arrhythmic sequences appeared consecutively, and two sequences of different tempi or pitches from the same original rhythm did not appear consecutively. Blocks were separated by a jittered silent period of 25–35 s (Figure 1E), and the 60 blocks lasted ∼52 min. The stimuli were presented during sleep at a comfortable listening level (∼70 dB) using a speaker placed at the infant’s foot level.

The hemodynamic brain response to the auditory stimuli was recorded using a frequency-domain based optical imaging system (Imagent; ISS) at wavelengths 690 and 830 nm, similarly to previous studies in premature neonates^39^^,^^81^ with a sampling frequency of 10.1215 Hz. We developed a high density multi-distance patch for premature infants for acquiring oxygenated hemoglobin (HbO) and deoxygenated hemoglobin (HbR) concentration changes. On each hemisphere there were 8 detectors and 10 emitters (Figure 1F) resulting in a total of 39 and 40 channels on the left and right hemispheres, respectively, with emitter-detector distances between 15 and 40 mm. Each channel is considered as a path with a banana shape between the emitter and the detector, with the maximum depth occurring at the midpoint of the distance between them. The midpoint of the theoretical banana was regarded as the optimal position of the channel, as it represents the maximum depth of photon penetration. The patches covered temporal regions as well as a part of frontal, sensorimotor, and parietal, regions of each hemisphere. However, the use of fiber optic patches did not allow recording the supplementary motor area and the more dorsal regions of the motor system at the same time as the ventral parts. To minimize errors in region of interest (ROI) localization, we implemented the following measures: (1) we used a 35 wGA preterm infant head model from AtlasViewer toolbox in MATLAB,^80^ and (2) digitized the positions of the emitters and detectors to reconstruct the HbO and HbR signals over each channel, taking into account each distance between emitters and detectors.

### Quantification and statistical analysis

#### Data processing

All data preprocessing was conducted in MATLAB (R2022b) using Homer3 functions^82^ as well as in-house scripts. Intensity signals were converted into optical density signals, which were then band-pass filtered (0.03–0.5 Hz). The modified Beer-Lambert Law with an age-adapted differential path length factor^83^^,^^84^ was applied to the two wavelength signals (690 and 830 nm) to convert optical density signals into HbO and HbR concentration changes. First, channels with poor signal-to-noise ratio were detected visually and discarded (4 on the left and 6 on the right hemisphere). Next, any sample in any of the channels with an absolute value greater than 0.01 (Molar × mm) was detected and a 3s window around that sample was identified as a movement artifact and excluded from further analyses. If an artifact sample was detected in more than 10 channels, that sample was discarded from all the channels. Next, for each participant for each channel, the signals were z-scored across the entire experiment. 30-s epochs were taken for each trial, consisting of a 5 s baseline prior to and 25 s after the onset of the stimulus. Linear detrending and then baseline correction were applied to each trial. A trial was removed if (1) the z-scored value of any sample within the trial exceeded 3 (adapted from,^39^ (2) if the within trial gradient of concentration over steps of 0.4 s exceeded 1.5 (adapted from^78^), (3) if the area under curve after stimulus onset was below 10, and finally, (4) if a part of the trial was detected as movement artifact and removed during the previous preprocessing step before z-scoring. If there were more than 80% rejected trials for a channel in each condition, the channel was excluded for that condition. On average 4.8874 ± 1.7003 and 9.9091 ± 1.8141 channels, from the left and right hemispheres respectively, per participant were discarded after the preprocessing steps (excluding visual inspection). Two participants were discarded from further analysis due to poor probe positioning, which resulted in low signal to noise ratio (*n* = 1), and due to a low number of remaining trials after preprocessing (*n* = 1), resulting in 11 participants for further analyses. The remaining trials over each channel were averaged over tempi and pitch variations for each participant in each condition (rhythmic and arrhythmic) to obtain the hemodynamic responses. A grand average across all participants was calculated for each channel, and channels with fewer than 4 participants contributing to the average were discarded from statistical analyses, leading to 33 (out of 39) and 27 (out of 40) and channels over the left and right hemispheres for further analyses. Subsequent analyses were conducted on the HbO signals, after downsampling to 1 Hz.^39^ In order to investigate the response dynamics, we computed the rising slope of the HbO average response for each condition (rhythmic and arrhythmic), at each channel location and for each participant using

Equation 1 Slope = ΔHbO/Δtime = (HbO_peak_ – HbO_stim.onset_) / (time_peak_ – time_stim.onset_)

To visualize the difference in the slope of HbO responses between rhythmic and arrhythmic conditions, we normalized the slope over each channel for each participant,

Equation 2 Slope_normalized_ = (Slope_RC_ – Slope_AC_) / (Slope_RC_ + Slope_AC_)

where RC and AC correspond to rhythmic and arrhythmic condition, respectively.

#### Statistical analysis

In order to compare the HbO responses corresponding to the rhythmic and arrhythmic conditions with the baseline at each channel location, we performed a temporal nonparametric cluster-based permutation (5000 permutations) on each channel as implemented in the FieldTrip toolbox,^85^ and contrasted the normalized amplitude with the average baseline value at each channel location. No spatial permutation was performed at this step as the objective was to evaluate whether the response at each channel location was significantly above the baseline. The initial threshold and final significance threshold were set to *p* < 0.05. To compare the HbO responses corresponding to rhythmic and arrhythmic conditions, we conducted a second nonparametric cluster-based permutation test (5000 permutations) at each channel. The initial threshold for cluster definition was set to *p* < 0.05 and the minimum number of neighbors to 1. The final significance threshold for summed *t* values within clusters was also set to *p* < 0.05. To compare the slope of the HbO signal between rhythmic and arrhythmic conditions we first performed a paired t-test on each channel, followed by a spatial cluster-based permutation test. The minimum number of neighbors was equal to 1. The final significance threshold was set to *p* < 0.05.

In order to compare the HbO response over the auditory regions with that over the sensorimotor and premotor regions, we calculated the average response over the time window 8–12 s (average over a 4 s window around the peak, over each channel and then averaged over channels belonging to the same ROI), over three ROIs (Figure 2) over each hemisphere, for each participant. Mirrored regions were selected in both hemispheres, and channels that remained after preprocessing and fell within the defined region were marked. This led to slight differences between the channels over the left and right hemisphere ROIs. We conducted a three-way repeated measure analysis of variance (rANOVA) with factors condition (rhythmic and arrhythmic), ROI (sensorimotor, auditory, and premotor) and hemisphere (left, right). Finally, we completed this analysis by performing two separate two-way rANOVA for each of the rhythmic and arrhythmic conditions, with factors ROI and hemisphere; and three separate rANOVA for each ROI with factors condition and hemisphere. Post-hoc ROI comparisons were performed using Tukey-Kramer correction for multiple comparisons.
