# Supplementary material for: Neural encoding of auditory rhythm beyond cortical auditory areas before the age of term
Source: iScience. 2025 Jul 24;28(9):113028. doi: 10.1016/j.isci.2025.113028 (PMC12390951; doi:10.1016/j.isci.2025.113028)
Supplement: Document S1. Figures S1 and S2 and Tables S1 and S2 [file mmc1.pdf]

iScience, Volume *28*

Supplemental information

Neural encoding of auditory rhythm beyond cortical auditory areas before the age of term

Ali Rajabi Mashhadi, Fabrice Wallois, Mohammadreza Edalati, Florence Levé, Alexandros Stamatiadis, Christelle Chazal, Laurel Trainor, and Sahar Moghimi

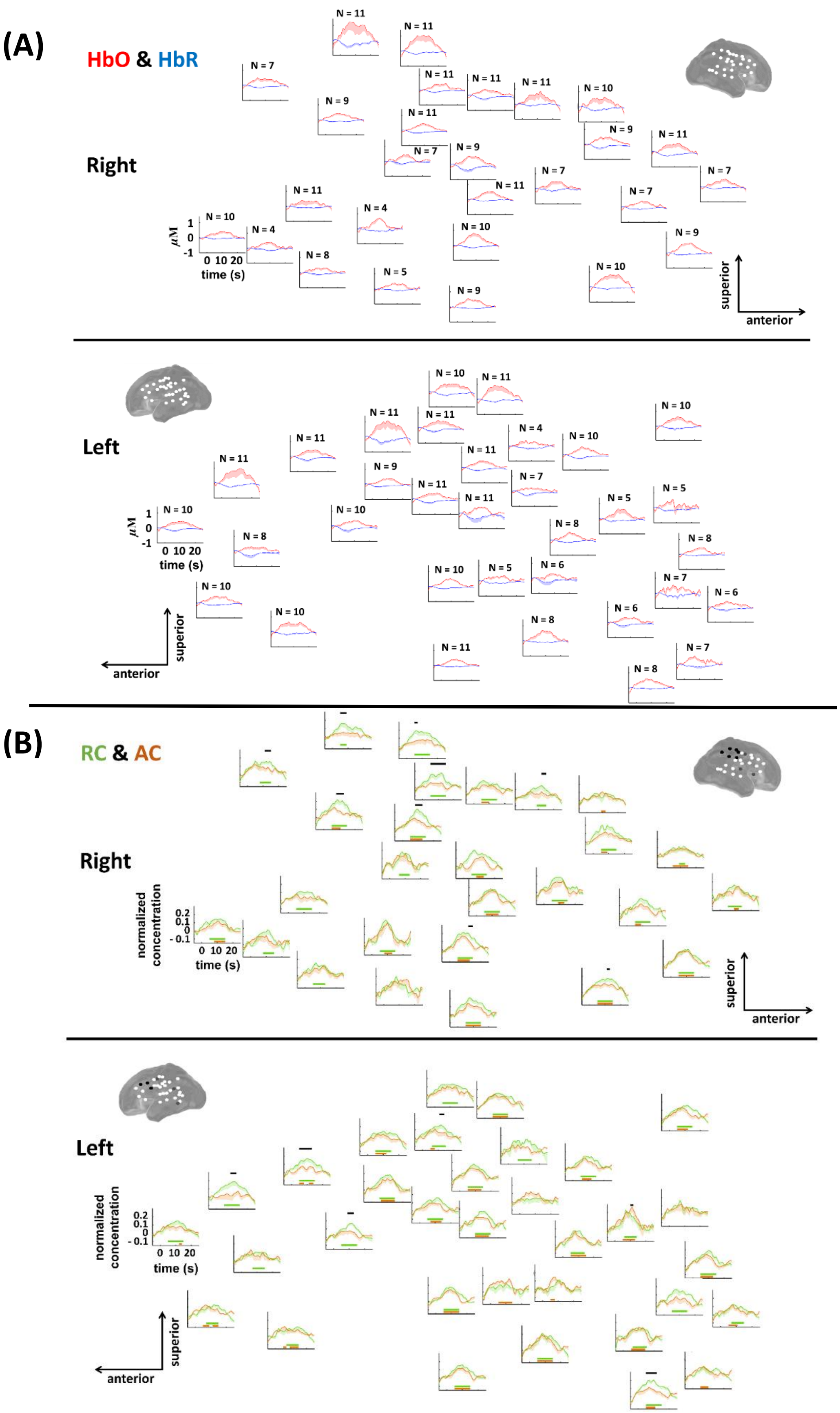
(A)
HbO & HbR
Right
Left
μM
time (s)
superior
anterior
(B)
RC & AC
normalized concentration

Figure S.1. Grand average time courses of hemodynamic responses across channels and conditions with statistical comparisons. (A) Time course of the grand average of HbO (red) and HbR (blue) across all conditions at each channel location. The *y* axis displays the concentration changes in μM. The *x* axis represents the time in seconds. Time zero indicates stimulus onset. N: number of participants contributing to the grand average responses in each channel. (B) Time course of the grand average of HbO for RC (green) and AC (orange) at each channel location. The *y* axis displays the concentration changes in arbitrary unit (a.u.). The *x* axis shows time in seconds, with zero marking the stimulus onset. The black bars indicate the time windows during which the RC differs significantly from the AC. The green bars indicate the time windows during which the RC differs significantly from its baseline, whereas the orange bars indicate the time windows during which the AC differs significantly from its baseline. All statistical analyses were performed with the cluster-based permutation test separately on each channel. The number of participants contributing to the grand average responses in each channel, is the same as the numbers indicated in (A).

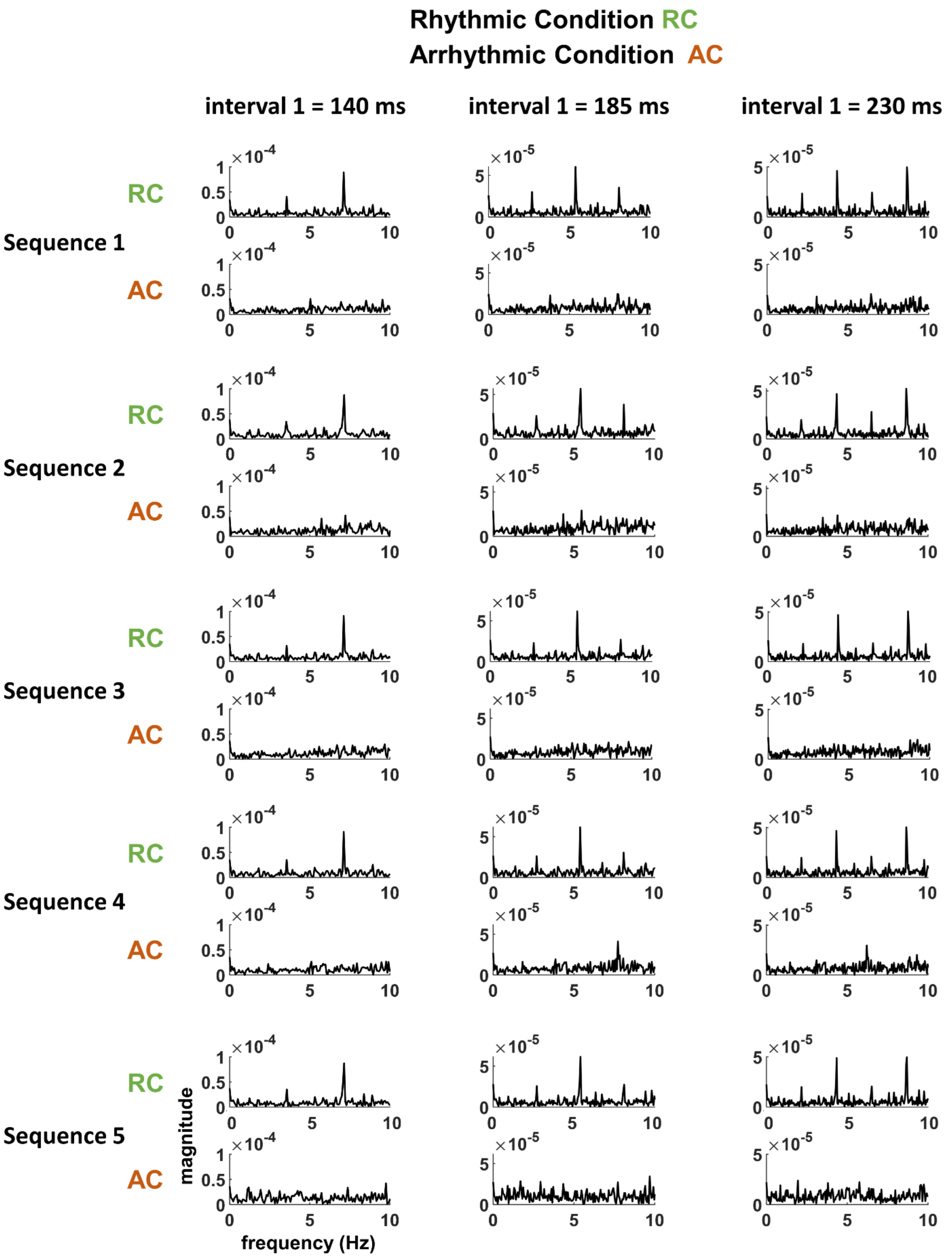

Figure S.2. Spectral content of rhythmic and arrhythmic stimuli. Power spectra of all stimuli for RC (Rhythmic Condition) and its AC (Arhythmic Condition) counterpart at the three tempi (interval 1 = 140, 185 and 230 ms).

Table S.1. Participant information. F: female, M: male, wk: week, d: day

Infant no.	Sex	GA at birth (wk+d/7)	GA at test (wk+d/7)	Birth weight (g)	Apgar (1 min)	Apgar (5 min)	Delivery
1	F	33 3/7	35 5/7	1360	1	3	Cesarian
2	F	32 5/7	34 6/7	1950	10	10	Vaginal
3	F	32 5/7	34 6/7	1830	10	10	Vaginal
4	M	28 2/7	35 3/7	1160	10	10	Vaginal
5	M	33 1/7	36 2/7	1800	10	10	Vaginal
6	M	33 1/7	36 2/7	1780	10	10	Vaginal
7	M	27 3/7	35 5/7	1200	10	10	Vaginal
8	F	33 6/7	35 2/7	1930	10	10	Vaginal
9	M	26 5/7	35 5/7	1020	8	10	Vaginal
10	F	28 4/7	40 6/7	610	3	5	Cesarian
11	M	30 1/7	36 3/7	1530	2	4	Vaginal
12	F	35 5/7	36 6/7	1900	10	10	Cesarian
13	M	34 3/7	35 2/7	2260	10	10	Vaginal

Table S.2. Rhythmic and corresponding arrhythmic sequences of all stimuli. Interval 1 = 140,185, and 230 ms (3 tempi for each rhythm). All other intervals in that sequence are multiplied by length chosen for the 1 interval.

Condition	Sequence																																											
	Sequence 1 (42 Intervals)																																											
Rhythmic	2	2	4	1	3	4	2	2	1	1	2	2	1	1	2	2	4	2	1	1	3	1	1	3	4	3	1	2	2	1	1	2	2	1	1	4	2	2	2	1	1	4			
Arrhythmic	3.2	2	3	0.67	1.33	1.2	1.5	0.8	2.4	1	2	1.33	4	1.5	1.33	1.33	1.2	4.8	0.8	2.5	0.8	2	0.67	2.5	2	1.5	1.33	1	0.75	2.67	1	3	1.2	2	5.33	3	4	2	4	2.67	1.5	2.25			
	Sequence 2 (43 Intervals)																																											
Rhythmic	4	2	2	1	1	2	2	2	4	1	3	3	1	4	1	1	1	1	2	1	1	4	2	2	3	1	4	2	2	1	1	2	3	1	2	2	1	1	2	2	1	1	4		
Arrhythmic	2	0.67	5	0.8	1.5	2.5	1	1.33	2	4	0.8	3	1.5	1.5	0.8	2.5	2.4	2	3.2	1	2	1	4	2.67	0.8	2.5	2	4	1.2	1.6	1.2	1	5	2	0.75	1.2	3	1.2	1.5	1.5	0.75	1.2	3.6		
	Sequence 3 (43 Intervals)																																											
Rhythmic	4	2	2	1	1	2	3	1	4	1	1	1	1	3	1	4	2	2	1	1	2	3	1	1	3	3	1	1	3	2	2	4	3	1	2	2	1	1	2	2	1	1	4		
Arrhythmic	3	0.75	2.67	1.2	3	1.33	1.2	0.8	5	2.4	2	1.2	2.5	0.8	0.8	2.25	2.4	2	2	1.2	2.25	1.5	4	1	3	0.8	1.33	1.33	1.5	1	2	1.5	0.67	3.2	3.75	0.8	1.5	0.8	3.6	1.6	3	1	5		
	Sequence 4 (43 Intervals)																																											
Rhythmic	2	1	1	3	1	1	3	2	2	4	1	3	3	1	2	2	1	1	2	4	1	1	1	1	3	1	2	1	1	2	2	4	3	1	4	2	2	2	2	2	1	1	4		
Arrhythmic	1.33	1.5	2	0.75	0.67	2	1.33	1.5	1	2	3.2	1	1.5	1.33	5	0.67	2.4	2.5	1	5.33	2	3.75	1	1.33	2.67	2	1.33	0.75	2	0.75	0.67	0.67	3.75	3.75	2	1.33	2.67	2.67	1.6	1.33	2.5	2.67	2		
	Sequence 5 (45 Intervals)																																											
Rhythmic	4	1	1	1	1	3	1	4	2	2	1	1	2	4	3	1	2	2	3	1	4	1	1	1	1	2	1	1	4	2	2	1	1	2	3	1	2	2	1	1	2	2	1	1	4
Arrhythmic	2.5	1.5	1	2	1	0.67	4.8	1	2	1.33	2.67	1.5	0.67	1.33	2.67	3	1	2	2	1	1	1	1	2	1	2	1	1.6	2.5	4	5	5	1	1	0.67	3.6	1	1	3	3	0.67	0.67	2.67	0.67	1.5
